# Knowledge of fertility period among reproductive age women in Kenya: a multilevel analysis based on 2022 Kenyan demographic and health survey

**DOI:** 10.1186/s40834-024-00287-7

**Published:** 2024-05-24

**Authors:** Bezawit Melak Fente, Yordanos Sisay Asgedom, Zufan Alamrie Asmare, Tirusew Nigussie Kebede, Beyene Sisay Damtew, Tadesu Wondu Workneh, Muluken Adamu Beyene, Beminate Lemma Seifu

**Affiliations:** 1 Department of General Midwifery, School of Midwifery, College of Medicine & Health Sciences, University of Gondar, Gondar, Ethiopia; 2 Department of Epidemiology and Biostatics, College of Health Sciences and Medicine, Wolaita Sodo University, Soddo, Ethiopia; 3 Department of Ophthalmology, School of Medicine and Health Science, Debre Tabor University, Debre Tabor, Ethiopia; 4 Department of Midwifery, School of Nursing and Midwifery Asrat Woldeyes Health Science campus, Debre Berhan University, Debre Berhan, Ethiopia; 5 Department of Midwifery, College of Health Sciences, Arsi University, Asella, Ethiopia; 6 Department of General Midwifery, School of Midwifery, College of Medicine and Health Sciences, University of Gondar, Gondar, Ethiopia; 7 Department of Midwifery, College of Health Sciences, Assosa University, Assosa, Ethiopia; 8 Department of Public Health, College of Medicine and Health Sciences, Samara University, Semera, Ethiopia

**Keywords:** Knowledge of fertility period, Reproductive age women, Kenya, Multilevel analysis, Fertility

## Abstract

**Background:**

Knowledge of the fertility period aids women in refraining and engaging in sexual intercourse to avoid and to get pregnant, respectively. The effect of community-level factors on knowledge of the fertility period was not yet known in Kenya. Therefore, we aimed to investigate the community- and individual-level determinants of knowledge of fertility period among women of childbearing age in Kenya.

**Methods:**

The 2022 Kenyan Demography and Health Survey data was used for the current study. This study included 16,901 women of reproductive age. To account for the clustering effects of DHS data and the binary nature of the outcome variable, a multilevel binary logistic regression model was applied. An adjusted odds ratio with a 95% confidence interval was reported to declare the statistical significance. In addition, the model that had the lowest deviance was the one that best fit the data.

**Results:**

The overall prevalence of knowledge of the fertility period among Kenyan women was 38.1% (95%CI = 37.3, 38.9). Women’s age, women’s education status, heard FP, contraceptive use, media exposure, and distance from health facility significant individual factors while place of residence, and community-level education, were all of factors were found to be strongly associated with knowledge of fertility period.

**Conclusion:**

As per the findings of our study, Knowledge of the fertility period among reproductive women was low in Kenya. In the era of increasing refusal of hormone-based family planning, fertility-awareness-based family planning methods may be an option. Promoting the correct fertility period through education and media outreach may be helpful strategies for enhancing fertility decision-making.

## Introduction

Knowledge of the fertility period is the knowledge about the possibility of conceiving during the menstrual cycle [1]. It is essential to determining the likelihood of conception [2, 3]. The women who had correct knowledge of the fertility period were assessed by when women think the fertile period is at the middle of the menstrual cycle [4]. The correct knowledge of the fertility period is important for women of reproductive age, both for planning conception or avoiding unintended pregnancies and unsafe abortions [5]. A lack of correct knowledge of the fertility period in sexually active women is likely to lead to unintended pregnancies in the absence of modern contraceptive use [6].

A woman who accurately determines when she is fertile (ovulating) will not become pregnant unintentionally or against her will [2]. Likewise, women who do not utilize contraception and are unaware of their fertile period are more likely to become pregnant unintentionally [6]. Reported side-effects of modern contraceptives are linked to discontinuation [7], and non-use those because of unmet need or fear of side effects [8, 9]. knowledge about the fertility period in reproductive women is necessary [10]. Enhanced knowledge of fertility can positively affect women’s reproductive life planning and timing of conception. For example, a woman’s knowledge about fertility may influence her desired age for childbearing and conception planning [10, 11].

Rates of knowledge of fertility period in Asian countries in which DHS was 20%, rates in South America was 25% [12], and in the United States (16%) [13]. In a study conducted in West Africa 38.8% [14] and in 29 African countries 15.5% [14] showed women had knowledge about the fertility period. Other studies conducted in Nigeria (25%) [15], Haiti (24.6%) [16], and low-income African countries (24.04%) [17] were revealed different result. The Knowledge of the fertility period among married women of reproductive age in Kenya was 25.5% recognized as a fertile window halfway between two periods [14].

Fertility changes throughout the woman’s reproductive years and many factors, such as lifestyle, age, and certain diseases, can affect fertility [18, 19]. Several studies have explored community and individual levels factors associated with correct knowledge fertility period among women of childbearing age. These factors include women’s age, place of residence, region, religion, women’s education, husband/partner’s education, occupational status, wealth index, marital status, contraceptive use, and pregnancy. Further, existing studies revealed that exposure to mass media family planning messages and being visited by fieldworkers are positively associated with women’s adequate knowledge fertility period [5, 13–18, 20–28].

To develop effective prevention programs for the region, it is important to have a clear understanding of the potential factors for women’s knowledge of the fertility period among women in Kenya. While there is evidence to support the importance of considering various exposures to the fertility period as a top priority for maternal and child health, there have been very few studies conducted in Kenya that specifically examine the factors related to knowledge of the fertility period among women of reproductive age [14, 22]. Those studies missed important variables like media exposure, which is crucial for the provision of information, and only examined individual-level factors, not even using multilevel analysis, which is appropriate for hierarchical data. In this study, national DHS data of Kenya was used that show variables at the individual and community level. Knowing the community-level components in addition to the individual-level ones makes it easier to incorporate interventional techniques. With regard to these considerations, this study aimed to identify the factors for knowledge of the fertility period among reproductive-age women. It will be essential to identify these determinants to develop evidence-based programs in Kenya, specifically targeting the significant factors.

## Methods

### Data source

We used the recent Kenya Demographic and Health Survey (2022) data after a reasonable request from the Measure DHS program [4, 29] available at (https://dhsprogram.com/Data/terms-of-use.cfm). Kenya Demographic and Health Survey (KDHS) was the seventh survey undertaken in Kenya, preceding similar surveys. The 2022 Kenya Demographic and Health Survey (KDHS) utilized a two-stage stratified sampling design. In the first stage, 1,692 clusters were selected from the Kenya Household Health Survey Framework (KHHSF) using the Equal Probability Selection Method (EPSEM). The survey includes multiple datasets for men, women, children, births, and households. We used the Individual Record dataset (IR file) for this study. Reproductive-age women from Kenya’s population were selected as the source, while those from designated EAs were chosen as the study population. A total weighted sample of 16,901 reproductive-age women was considered for the final analysis.

### Study variables

#### Outcome variable

The outcome variable for this study was women’s correct knowledge of the fertility period (KOC). In the DHS, the question on KOC answered by women of reproductive age was “When is the ovulation time?” Response options were: “during her period”, “after the period ended”, “middle of the cycle”, “before the period begins”, “at any time”, and “don’t know”. The outcomes variable was recoded and all respondents who indicated “middle of the cycle” were considered as correct knowledge of the fertility period and coded as “1”, and the other responses, incorrect knowledge of fertility period, were coded as “0” [14, 16, 28].

#### Explanatory variables

Explanatory factors included women’s age in years (15–19, 20–24, 25–29, 30–34, 35–39, 40–44, 45–49), women’s educational level (no formal education, primary school, secondary and above), husband’s educational level (no formal education, primary school, secondary and above), marital status (not married, married), working status (not working, working) and parity (0, 1–2, 3–4, 5+), media exposure[(newspaper, radio, or television (TV)] was assessed in terms of frequency (no, yes), wealth index (poorest, poorer middle, richer and richest), distance to health facility (big problem, not a big problem), place of residence (urban, rural), community literacy level (low, high), community poverty level (low, high) and community media exposure level (low, high).

### Statistical analyses

Descriptive analysis was performed using frequency and percentage distributions to examine the characteristics of respondents and knowledge of the fertility period. This was followed by bivariate multilevel logistic regression to select variables that had a significant association with knowledge of fertility period at a p-value less than 0.25. A multicollinearity test was performed using variance inflation factor (VIF) for all statistically significant variables at the bivariate multilevel logistic regression. Using the multilevel logistic regression (MLLR) method, we created four different models to assess whether the individual/household and community-level factors had significant associations with the outcome variable (knowledge of fertility period). The first model was a null model (Model 0), which had no explanatory variables and it showed variance in the knowledge of the fertility period. The second model (model I) comprised individual/household-level factors and the third model (Model II) comprised community-level factors. The last model, (Model III), was the complete model that included factors at both the individual/household and community levels.

All four MLLR models included fixed and random effects [30, 31]. The fixed-effect model showed the association between the explanatory variables and the outcome variable, and the random effects signified the measure of variation in the outcome variable based on PSU, which was measured by Intra-Cluster Correlation (ICC) [32]. The model ft. was assessed using the Akaike’s Information Criterion (AIC) [33]. We used the “melogit” command to run the MLLR models. The analyses were performed using Stata version-14 software (Stata Corp, College Station, Texas, USA). We also followed the guidelines for Strengthening Observational studies in Epidemiology (STROBE) [34].

## Results

### Background characteristics of respondents

A total of 16,901 women who had given birth within 5 years preceding the survey were included in this study. Most women (61.4%) lived in rural areas, and half of women (51.2%) had attained secondary and above education. About 3339(19.7%)women were found in the age groups of 15–19 years followed by the age groups of 20–24 years 3062(18.1%). The majority of women (81.0%) had media exposure, two-thirds (65.3%) reported contraceptive utilization and nearly two-thirds (65.3%) of women heard about family planning **(**Table 1**)**.

**Table 1:** Distribution of the study population by socio-demographic and reproductive-related characteristics in Kenya DHS 2022(*n* = 16,901)

Variables	Weighted frequency (%)	Knowledge of fertility period	
		Do not know	Know
**Age**			
15–19	3339(19.7)	2418	921
20–24	3062(18.1)	1850	1212
25–29	2833(16.7)	1645	1188
30–34	2400(14.2)	1457	943
35–39	2289(13.5)	1372	917
40–44	1637(9.7)	939	698
45–49	1341(7.9)	779	562
**Marital status**			
Not married	5259(31.1)	3416	1843
Married	11,642(68.9)	7044	4598
**Education**			
no education	2075(12.3)	1468	607
primary	6171(36.5)	4166	2005
Secondary and above	8655(51.2)	4826	3829
**sex of household head**			
Male	10,231(60.5)	6464	3767
Female	6670(39.5)	3996	2674
**wealth status**			
Poorest	3758(22.2)	2652	1106
Poorer	2975(17.6)	1909	1066
Middle	3308(19.5)	2072	1236
Richer	3753(22.2)	2212	1541
Richest	3107(18.4)	1615	1492
**Distance Health facility**			
big problem	4589(27.2)	2992	1597
not a big problem	12,312(72.8)	7468	4844
**Partner’s education level**			
No education	1395(8.3)	957	438
Primary	3595(21.3)	2338	1257
Secondary and above	11,911(70.4)	7165	4746
**Parity**			
No	6854(40.5)	4180	2674
1–2	9104(53.8)	5663	3441
3–4	929(5.5)	611	318
5+	14(0.1)	6	8
**Media exposure**			
No	3216(19.0)	2296	920
Yes	13,685(81.0)	8164	5521
**Contraceptive method**			
User	7015(41.5)	4125	2890
Non user	9886(58.5)	6335	3551
**Heard FP**			
No	5875(34.7)	4076	1799
Yes	11,026(65.3)	6384	4642
**Pregnancy desire**			
Have no desire	7512(44.5)	4607	2905
Have desire	9389(55.5)	5853	3536
**Occupation**			
Not working	7725(45.7)	4978	2747
Working	9176(54.3)	5482	3694
**Residence**			
Urban	6517(38.5)	3824	2693
Rural	10,384(61.4)	6636	3748
**Community education**			
High	499(29.5)	822	369
Low	1191(70.5)	375	124
**Community poverty**			
High	781(46.2)	623	286
Low	909(53.8)	574	207
**Community media**			
Low	849(50.2)	597	252
High	842(49.8)	601	241

*FP: Familiy planing

### Prevalence of knowledge of the fertility period

**Fig. 1:**
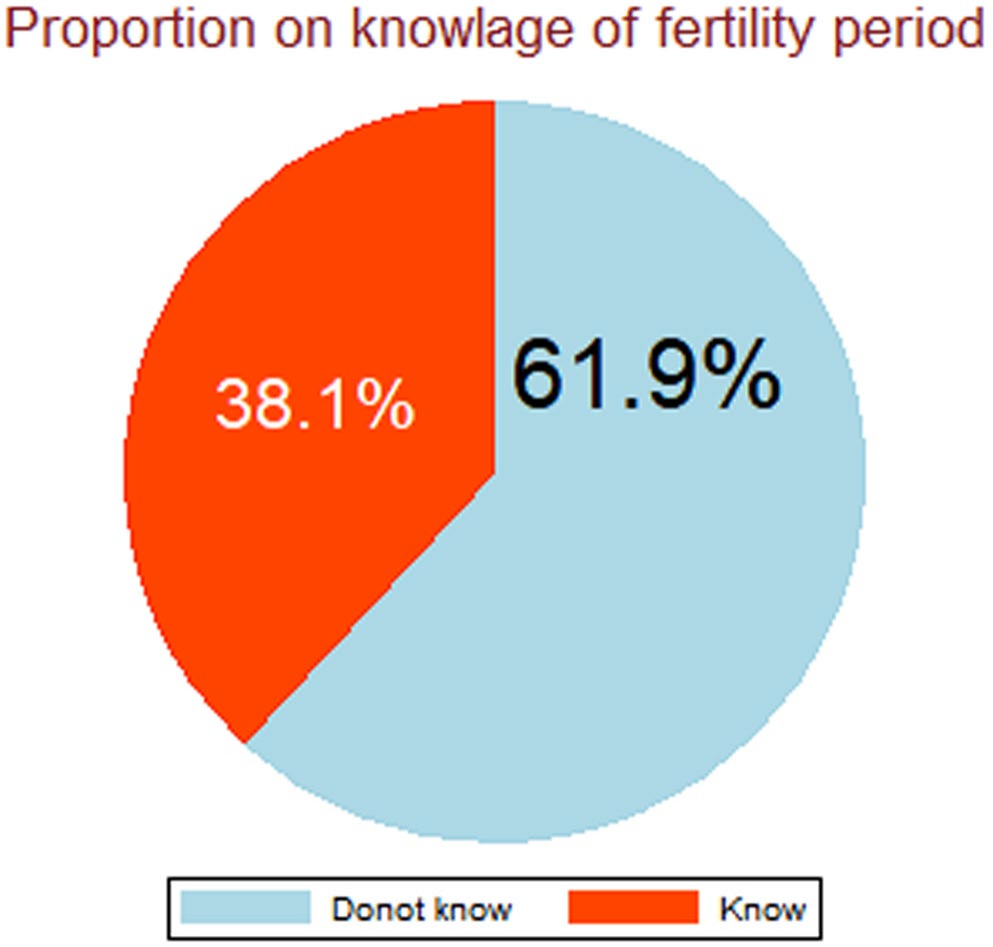
Proportion of knowledge of the fertility period

The prevalence of knowledge of the fertility period among women of reproductive age in Kenya was 38.1%( 95% CL: 37.3, 38.8%) **(**Fig. 1**).**

### Factors associated with high-risk fertility behavior among reproductive-age women in Kenya

In the multivariable mixed effect binary logistic regression model, women’s age, women’s education status, heard about FP, contraceptive use, media exposure, and distance from health facility significant individual factors while place of residence, and community-level education were found to be statistically significant factors from community-level factors of knowledge of fertility period among Kenyan reproductive age women.

The odds of good knowledge were 1.81 times higher in the age group of 20–24 years (AOR = 1.81; CI = 1.17, 2.81). Similarly, the odds were 1.97 times higher in the age group of 25–29 years (AOR = 1.97; CI = 1.17, 3.30); 2.17 times higher in the age group ranging from 30 to 34 years (AOR = 2.17; CI = 1.24, 3.80); 2.56 times higher in the age group of 35–39 years (AOR = 2.56; CI = 1.44, 4.56); 2.36 times higher in the age group of 40–44 years (AOR = 2.36; CI = 1.26, 4.43); and 3.65 times higher in the age group of 45–49 years (AOR = 3.65; CI = 1.92, 6.93) than the age group of 15–19 years **(**Table 2**)**.

**Table 2:** Multilevel analysis of factors associated with knowledge of fertility period among reproductive-age women in Kenya, 2022(*N* = 16,901)

Variables	Null model	Model I	Model II	Model III
**Age**				
15–19		1		
20–24		1.730(1.516,1.974)		1.81(1.17, 2.81)^*^
25–29		2.057(1.763, 2.400)		1.97(1.17, 3.30)^*^
30–34		1.990(1.686, 2.349)		2.17(1.24, 3.80)^*^
35–39		2.273(1.912, 2.703)		2.56(1.44, 4.56)^*^
40–44		2.578(2.141, 3.104)		2.36(1.26, 4.43)^*^
45–49		2.503(2.060, 3.041)		3.65(1.92, 6.93)^*^
**Occupation**				
Not working		1		
Working		1.869(1.796, 1.947)		1.27(0.97, 1.67)
**Education**				
No education		1		
Primary		1.120(0.944,1.32)		1.11(0.72, 1.71)
Secondary and above				1.53(1.00, 2.33)^*^
**Pregnancy desire**				
Have no desire		1		
Have desire		1.054(0.968, 1.149)		0 0.98(0.74, 1.30)
**Recently active sex**				
Not active		1		
Active		1.133(1.034, 1.242)		1.27(0.92, 1.75)
**Marital status**				
Single		1		
Married		1.011(0.896, 1.141)		1.31(0.81, 2.12)
**Wealth status**				
Poorest		1		
Poorer		1.154(1.010, 1.319)		1.03(0.628, 1.69)
Middle		1.111(0.969, 1.274)		0.74(0.46, 1.20)
Richer		1.221(1.057, 1.410)		1.19(0.72, 1.97)
Richest		1.496(1.272, 1.758)		1.63(0.92, 2.88)
**Heard FP**		1		
No				
Yes		1.255(1.150, 1.369)		1.56(1.18, 2.05)*
**Contraceptive use**				
User		1		
Non user		0.978(0.899, 1.064)		1.42(1.06, 1.89)*
**Media exposure**				
No		1		
Yes		1.359(1.205, 1.533)		1.53(1.09, 2.16)*
**Parent education**				
No education		1		
Primary		0.746(0.614, 0.906)		1.03(0.59, 1.79)
Secondary		0.874(0.722, 1.059)		1.39(0.78, 2.45)
**Distance health facility**				
Big problem		1		
Not a big problem		1.031(0.943,1.128)		1.55(1.14, 2.09)*
**Sex house head**				
Male		1		
Female		1.145(1.055, 1.24)		1.15(0.87, 1.52)
**Residence**				
Rural			1	1
Urban			1.11(0.79, 1.55)	1.79(1.17, 2.73)*
**Community level education**				
Low			1	
High			1.74(0.54,2.02)	1.44(1.04, 2.00)*
**Community level poverty**				
High			1	
Low			1.89(0.68, 2.15)	1.16(0.89, 1.53)
**Community Media**				
Low			1	
High			1.83(0.64,2.08)	1.21(0.93, 1.58)

*p-value < 0.05

The odds of correct knowledge of the fertility period were higher among women who attended secondary and above (AOR = 1.53 1.95; 95% CI: 1.00, 2.33) compared to those with primary level or less. The odds of having good knowledge of the fertility period among women who perceived the distance to the health facility as not a big problem was increased by 1.55 times (AOR = 1.55, 95% CI: 1.14, 2.09) than women who perceived the distance to the health facility as a big problem. The odds of hearing about family planning was 1.42 times (AOR = 1.42, 95% CI: 1.06, 1.89) more than women not heard about family planning. The odds of women erewere contraceptive users were 1.56 times (AOR = 1.56, 95% CI: 1.18, 2.05) more than women contraceptive non-users **(**Table 2**)**.

Regarding community-level factors, we found urban women had higher odds (AOR = 1.79; 95% CI: 1.17, 2.73) of having correct knowledge of the fertility period compared to rural women. Higher odds of knowledge of fertility period among women from high community-level education (AOR 2.24, 95% CI 1.32–3.81) compared to those from low community-level education **(**Table 2**)**.

### Random effects (measures of variations) results

The random effect models of the individual/household and community level factors associated with knowledge of the fertility period are shown in Table 3. We observed that the values of the AIC decreased and deviance across the models, indicating the best-fitted model was chosen based on the lowest deviance value (1857.5402) and AIC (12.10). The ICC in the null model (ICC = 18.8%) showed that the odds of knowledge of the fertility period varied across clusters (σ2 = 1.04, 0.85–1.29). The between-cluster variations decreased by 2.6% in the model I, from 18.84% in the null model to 16.24% in the model I. From Model I, the ICC decreased again by 2% in Model II (ICC = 14%) and then declined by 2% in the complete model (Model III, ICC = 12.1%. These estimates showed that the variations in the likelihood of knowledge of the fertility period can be attributed to the variances in the clustering at the primary sampling units **(**Table 3**)**.

**Table 3:** Random effect results for knowledge of the fertility period and its individual and community level factors: evidence from KDHS (*N* = 16,901)

Random effects	Null	M1	M2	M3
Log-likelihood	-10778.136	-10446.272	-986.5695	-928.77009
ICC (%)	18.84	16.24	14.00	12.10
AIC	21560.27	20942.54	1985.139	1915.54
BIC	21575.74	21135.92	2017.734	2073.082
Deviance	21556.272	20892.544	1973.139	1857.5402
PCV (%)	Ref	4.0	26.3	40.8
Wald chi‑square and p‑value	Ref	X^2^ = 614.33,, *p* < 0.001	X^2=^5.43, *p* < 0.001	X^2=^108.90, *p* < 0.001

*ICC: Intra-class correlation coefficient

* PCV: proportional change in variance

## Discussion

In this study, we investigated knowledge of the fertility period and its individual/household and community level factors among women of reproductive age using a Kenyan nationally representative dataset. According to the current study, 38.1% (95% CL: 37.3, 38.8%) of Kenyan women knew of the fertility period. It was consistent with studies reported in West Africa which showed that about 38.8% of women know [14] the period of ovulation to be halfway through the menstrual cycle. The overall magnitude of knowledge of the fertility period in the current study was higher than the findings in Ethiopia (23.6%) [20, 28, 35], Nigeria (25%) [15], Haiti (24.6%) [16], 29 African countries(15.5%) [14], low-income African countries (24.04%) [17], and United state (16%) [13]. On the other hand, the result of this study was lower than studies in Spain(51.7%) and the United States(57.5%) [24]. This disparity might be due to differences in the study period and study design, quality of maternal services utilization, and population included in the study difference. Furthermore, increased levels of education in particular for women and girls, increased urbanization, women’s empowerment and growing labor force participation, and expanded access to reproductive health-care services.

Age of the woman, women’s educational status, heard about FP, contraceptive use, media exposure, and distance from health facility significant individual factors while the place of residence, and community-level education were significantly associated with knowledge of the fertility period.

The odds of knowledge about the fertility period increased as the age of women increased. This finding is consistent with research from Ethiopia [28], the United States [24], and Spain [23], Low-Income African countries [17] which found that women who are at a later age in their reproductive lives had more precise awareness of their fertility period than those who are just starting. Given that age is a major educator in human life, the link between these two factors may be explained by repeated exposure and older women having greater experience with reproduction [21].

Women who attained secondary and higher education had better knowledge about the highest conception probability period compared to women not attending formal education. This finding is similar to reports of many studies like DHS report studies in Ethiopia [28, 35], African countries [14], Low-income countries [17], and another study [36]. This result might be due to the fact that formal education provides better opportunities for women to comprehend the science of the reproductive system. Nowadays, research on the effects of education on family planning techniques, including fertility awareness, has shown that education broadens people’s understanding of reproductive health [37].

Women who heard about family planning methods in media (like in a newspaper, TV, radio, and phone messages) in the last few months were more knowledgeable about their highest conception probability period than those who had no media exposure. This finding is supported by another study conducted in Low-income countries [17]. Similarly, women who had media exposure were more knowledgeable about their fertility period compared to their counterparts. This is supported by studies conducted in African countries and Ethiopia [14, 28]. The possible reason for this association might be due to obtaining information regarding both traditional and modern contraceptive methods through this media [14, 28].

Better knowledge of about fertility period was found in the women who are currently contraceptive users than those who are not in use which is in line with a study conducted in Ethiopia [28] and in low-income countries [17]. This might be due to women who use modern contraception having knowledge of their ovulatory cycle and using modern contraception to avoid unintended pregnancy so that women have a good awareness of it [17].

Women who lived in urban residences had better knowledge of the fertility period than rural residents. This finding is in agreement with studies conducted in Nigeria [15], Spain [23], Ethiopia [20, 28], and Africa [5, 17]. The reason for having better knowledge in urban residents might be because of the favorable conditions like better state of affairs for socioeconomic, educational skills, increased access to media, internet/websites, and better utilization of health care services [20, 28]. The cumulative effect provides better information associated with family planning and other reproductive health services among urban residents [5, 17].

### Strengths and limitations of the study

The strengths of this study incorporated; first, it was conducted using data from a large national survey which provides adequate power to detect the true effect of the independent variables. Second, the sampling weight was applied during the analysis to get reliable estimates and standard errors. Additionally, we were able to study correct knowledge of the fertility period by looking at two levels individual/ household and community which allowed us to study hierarchical or clustered structures that may influence outcomes. As a limitation, since the study used cross-sectional data, a causal relationship between knowledge of the fertility period and the identified independent variables cannot be established. The DHS relies on self-reported data and is subject to recall bias.

## Conclusion

Knowledge of the fertility period among reproductive-aged women was found to be low in Kenya in this study. Age of woman, women’s educational status, heard about FP, contraceptive use, media exposure, and distance from health facility significant individual factors while place of residence, and community-level education were found to be statistically significant factors from community-level factors of knowledge of fertility period among Kenyan reproductive age women. To increase levels of education in particular for women and girls, increased urbanization, women’s empowerment and growing labor force participation, and expanded access to reproductive health-care services including for family planning. Improving fertility awareness through comprehensive reproductive education or counseling could be one of the operational ways to control unintended pregnancy. Future researchers are also recommending addressing the missed independent variables using primary data.

### Implications of the findings

Knowledge of the fertility period is an important factor in fertility awareness and decision-making. This is especially important in the context of Kenya, characterized by low contraceptive prevalence and a high unmet need for modern contraception. Promoting correct fertility periods through education and media outreach may be helpful strategies for enhancing fertility decision-making and, subsequently, increasing contraceptive use, including modern methods, as well as fertility-based methods such as natural family planning methods, which depend on an accurate fertility period.

## Data Availability

Permission to get access to the data was obtained from the measure DHS program online request from http://www.dhsprogramcom.website and the data used were publicly available with no personal identifier.
